# Fabrication of a Microbial Biosensor Based on QD-MWNT Supports by a One-Step Radiation Reaction and Detection of Phenolic Compounds in Red Wines

**DOI:** 10.3390/s110202001

**Published:** 2011-02-08

**Authors:** Seul-Ki Kim, Hai-Doo Kwen, Seong-Ho Choi

**Affiliations:** Department of Chemistry, Hannam University, Daejeon 305-811, Korea; E-Mails: 332816@hanmail.net (S.-K.K.); hdkwen@naver.com (H.-D.K.)

**Keywords:** microbial biosensor, quantum dots, one-step radiation reaction, electron transfer supports, phenolic compounds, red wines

## Abstract

An *Acaligense sp.*-immobilized biosensor was fabricated based on QD-MWNT composites as an electron transfer mediator and a microbe immobilization support by a one-step radiation reaction and used for sensing phenolic compounds in commercial red wines. First, a quantum dot-modified multi-wall carbon nanotube (QD-MWNT) composite was prepared in the presence of MWNT by a one-step radiation reaction in an aqueous solution at room temperature. The successful preparation of the QD-MWNT composite was confirmed by XPS, TEM, and elemental analysis. Second, the microbial biosensor was fabricated by immobilization of *Acaligense sp.* on the surface of the composite thin film of a glassy carbon (GC) electrode, which was prepared by a hand casting method with a mixture of the previously obtained composite and Nafion solution. The sensing ranges of the microbial biosensor based on CdS-MWNT and Cu_2_S-MWNT supports were 0.5–5.0 mM and 0.7–10 mM for phenol in a phosphate buffer solution, respectively. Total concentration of phenolic compounds contained in commercial red wines was also determined using the prepared microbial immobilized biosensor.

## 1. Introduction

Recently, direct electrochemistry and catalytic activity of many biomolecules have been obtained at electrodes modified with nanomaterials, such as carbon nanotubes (CNTs) [1–3], gold nanoparticles [4–6], silica nanoparticles [7], and zirconium oxide nanoparticles [8]. Among them, CNTs have been one of the most actively studied electrode materials in the past few years due to their unique electronic and mechanical properties [9,10]. From a chemical point of view, CNTs are expected to exhibit inherent electrochemical properties similar to other carbon electrodes widely used in various electrochemical applications. CNTs, however, show very different electrochemical properties compared to those of other carbon-based nanomaterials, such as C_60_ and C_70_ [11]. The sensitivity of the electronic properties of CNTs has been widely used for various electrochemical sensors as an electron transfer mediator or an electrode support [12,13]. CNT-modified electrodes have been developed in order to use them as biosensors [14–16]. Most of these methods involve the immobilization of enzyme molecules on the electrode surface, such as CNT-composite electrode [17], vertically aligned nanotube electrode arrays [18], layer-by-layer electrodes [19], and CNT-coated electrodes [20]. CNT-coated electrodes can be easily manufactured as long as stable CNT suspensions are maintained.

In order to improve the efficiency of electron transfer between the redox center of enzyme and the electrode, many nanomaterials have been used [21–25]. Owing to their unique properties, quantum dots (QDs) have generated considerable interest as electron transport nanomaterials for biodetection. Huang *et al.* [26] reported the direct electron transfer of glucose oxidase (GOD) adsorbed on a CdS QD modified pyrolytic graphite electrode, where the enzyme demonstrated significantly enhanced electron-transfer reactivity. The CdS QD-coated electrode displayed a pair of well-defined redox peaks for GOD. Huang *et al.* [27] also used CdSe-ZnS QD as an electron transport material for preparing biosensors. On the other hand, a CdS QD modified SiO_2_ nanoparticle was synthesized by γ-ray irradiation in order to obtain photovoltatic effect [28]. The CdS QD was well dispersed on the surface of the SiO_2_ nanoparticles due to its hydrophilic properties. However, little work has been reported on the deposition of QDs onto CNT surfaces with hydrophobic properties using gamma-ray irradiation. To our knowledge the QD-CNT composite has not been applied as an electron transport material and immobilization support for preparing microbial biosensors until now.

Wines, particularly red wines, contain numerous biologically active compounds, the most important of which are phenolic compounds. The nutritional importance of phenolic compounds is attributed to their antioxidant properties. In particular, flavonoids and related phenolic compounds which are naturally found in red wines have gained increasing interest [29]. Red wines have been reported to be preventive of many ailments, and they play a possible role in reducing thrombotic and anthrogenic processes. Phenolic compounds also contribute substantially to the quality of wines and affect their color, flavor, stability and aging behavior [30]. However, little has been reported regarding the determination of total amount of phenolic compounds in red wine. This can be easily determined via electrochemical methods.

In this study, we synthesized QD-MWNT composites, CdS-MWNT and Cu_2_S-MWNT, to immobilize microbes by a one-step radiation reaction in an aqueous solution. The microbial biosensor was then prepared by immobilization of *Acaligense sp.* which is known as a potent phenol removing bacteria [31] on to QD-MWNT composite electrode, which was prepared by hand-casting onto a GC electrode surface. The prepared microbial biosensor was evaluated by its sensing efficiency for phenol in a phosphate buffer solution. Total phenolic compounds in two commercial red wines were also examined using the prepared microbial biosensor.

## 2. Experimental Section

### 2.1. Reagents

*Acaligenes sp.* (1.0 × 10^9^ CFU/mL) was obtained from Scientec Lab Center Co., Ltd. (Daejeon, Korea). Analytical cadmium sulfate (3CdSO_4_·8H_2_O), copper sulfate (CuSO_4_) and sodium thiosulfate pentahydrate (Na_2_S_2_O_3_·5H_2_O) as sulfur sources were obtained from Junsei Chemical Co., Ltd. (Kyoto, Japan). Vinylphenyl boronic acid (VBA), phenol, *p*-chresol, catechol, gallic acid, and Nafion^®^ solution were of analytical reagent grade (Sigma-Aldrich, Korea) and were used without further purification. MWNTs (CM-95) were supplied by Hanwha Nanotech Co., Ltd. (Korea). Solutions for the experiments were prepared with water purified in a Milli-Q plus water purification system (Millipore Co. Ltd., USA, the final resistance of water was 18.2 MΩ cm^−1^) and were degassed prior to each measurement. Other chemicals were of reagent grade. The red wines tested for phenolic compounds were Amor (made in Chile) and Blue Nun (made in Germany).

### 2.2. Fabrication of the Microbial Biosensor Based on QD-MWNT Composite

**Scheme 1.**
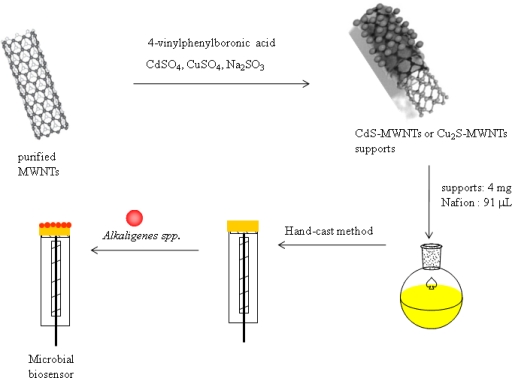
Preparation procedure of the electrochemical microbial biosensor based on QD-MWNT supports by a one-step radiation reaction.

Scheme 1 exhibits the preparation procedure of the electrochemical microbial biosensor based on QD-MWNT supports by a one-step radiation reaction. In order to use the supporting materials for the biosensor, the MWNTs were firstly purified by treatment with phosphoric acid at 50 °C for 5 h to remove the catalyst and non-crystallized carbon impurities. The purified MWNTs were then used as supporting materials for the deposition of QDs. In detail, the MWNTs (3.0 g), VBA (2.0 g) as an anchoring agent, CdSO_4_ (0.001 mol) as a Cd^2+^ source, and Na_2_S_2_O_3_ (0.001 mol) as a S^−2^ source were dissolved in H_2_O (182 mL) and 2-propanol (18.0 mL) was then added as the radical scavenger to improve the yield of nanocomposite. Nitrogen gas was bubbled through the solution for 30 min to remove oxygen gas, and the solution was irradiated by γ-ray from a Co-60 source under atmospheric pressure and an ambient temperature. A total irradiation dose of 30 kGy (a dose rate =1.0 × 10^4^ Gy/h) was used. The obtained samples was separated by centrifuge with 2,000 rpm, and then dried in a vacuum oven at 50 °C for 18 h. The Cu_2_S-MWNT composite was also prepared as the same method described above.

In order to immobilize QD-MWNT on to the GC electrode, the mixed solution was prepared using 5% Nafion^®^ solution (91 μL) and QD-MWNT support (4.0 mg) by stirring for 24 hours, then the mixed solution (10 μL) was coated on the surface of a pre-cleaned GC electrode (0.02 cm^2^) by the hand-cast method. The *Alkaligenes spp.*-immobilized biosensor was then fabricated by immobilization of 10 μL of *Alkaligenes spp.* (1.0 × 10^9^ CFU/mL) onto the QD-MWNT-coated electrode. The prepared *Alkaligenes spp.*-immobilized biosensor was dried for 1 hour at room temperature and was kept at 4 °C until use.

### 2.3. Instrumentation

Cyclic voltammetric experiments were performed with a Potentiostat/Gavanostat model 283 (Ametek PAR, USA). All experiments were carried out with a conventional three-electrode system. The working electrode was the GC electrode coated with the QD-MWNT composite. The counter electrode was the platinum wire, and the reference electrode was Ag/AgCl (sat’d KCl). The surface morphology of the samples was determined by HR-TEM (JEOL, JEM-2010, USA). The metal atom content of the samples was analyzed using an inductively coupled plasma-atomic emission spectrometer (ICP-AES) (Jobin-Yvon, Ultima-C, USA). The X-ray photoelectrons spectra of the samples have been obtained using ESCALab 220i (VG Scientific) equipped with a full 180° hemispherical electrostatic analyzer to examine the chemical state of the constituent elements. As a phonon source, Al Kα radiation (1,486.6 eV) was used. The half-width at half-maximum of the 4f_7/2_ line in the XPS spectrum of gold obtained at our XPS spectrometer was smaller than 1.0 eV. The energy scale of the spectrometer was calibrated using the lowest BE component of C 1 s peak (285.0 eV). The C 1 s spectra were deconvoluted using a Gaussian-Lorentzian model to obtain the best binding energy values.

## 3. Results and Discussion

### 3.1. Synthesis and Characterization of QD-MWNT Supports by a One-Step Radiation Reaction

**Figure 1.**
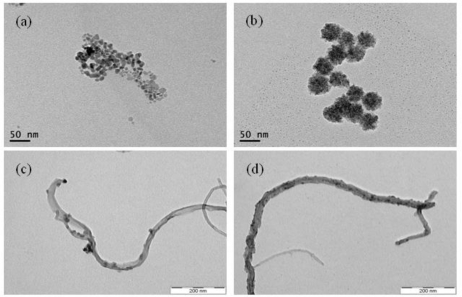
Transmission electron microscopy images of the CdS QD **(a)**, Cu_2_S QD **(b)**, CdS-MWNT support **(c)** and Cu_2_S-MWNT support **(d)** prepared by γ-irradiation.

As shown in Scheme 1, we synthesized polymer-stabilized QDs and QD-MWNT supports for microbial biosensors by a one-step radiation reaction. Figure 1 shows the TEM images of CdS QD (a), Cu_2_S QD (b), CdS-MWNT (c), and Cu_2_S-MWNT supports (d) for a microbial biosensor prepared by γ-irradiation in an aqueous solution at room temperature. Spherical CdS and Cu_2_S QDs with a uniform size were formed [Figure 1(a,b)]. In the case of the Cu_2_S QD, much smaller particles are aggregated into secondary particles due to their much smaller dimensions and higher surface energy, as shown in Figure 1(b).

In a previous paper [32], we prepared CdS nanoparticles and CdS-polyacrylonitrile nanocomposites and discussed the mechanism of the formation of CdS QD nanoparticles by γ-irradiation in an aqueous solution. In this experiment, we have used poly(vinylphenylboronic acid), PVBA, as a stabilizing agent in order to stabilize QD nanoparticles in an aqueous solution. As shown in Figure 1(a,b), we successfully prepared the PVBA-stabilized QD nanoparticles, however these QD nanoparticles couldn’t be used as supporting materials for a microbial biosensor because of their aggregation. Therefore, we deposited the QD nanoparticles onto a MWNT surface using γ-ray irradiation in an aqueous solution as shown in Figure 1(c,d). We used the VBA as an anchoring agent since the MWNT surface has hydrophobic property, while QD nanoparticles have hydrophilic properties. For immobilization of the QD nanoparticles onto MWNT surface with hydrophobic property, we need the anchoring agents such as vinyl monomers, which possess a hydrophobic site and a hydrophilic site in an aqueous solution. In previous studies, we successfully deposited the Pt-Ru nanoparticles onto MWNT surface using various anchoring agents by γ-ray irradiation [33–35]. As shown in Figure 1(c,d), the CdS QD (∼20 nm in size) and Cu_2_S QD (∼7 nm in size) were well dispersed onto MWNT surface. This QD-MWNT nanocomposite can be used for electron transport nanomaterials and biomolecule immobilization nanomaterials.

**Figure 2.**
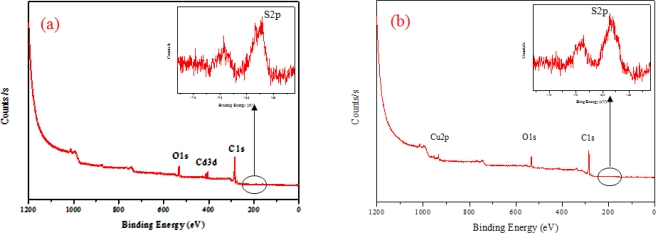
X-ray photoelectron spectroscopy spectra of the CdS-MWNT **(a)** and Cu_2_S-MWNT **(b)**.

Figure 2 shows the X-ray photoelectron spectroscopy spectra of the CdS-MWNT (a) and Cu_2_S-MWNT (b) supports for a biosensor. The peaks at 294.8 and 532.2 eV are attributed to C and O, corresponding to MWNT and functional groups like carboxyl group, carbonyl group, and hydroxyl group formed on the surface of MWNT by acid treatment. The Cd_3d_ and S_2p_ peaks at 406 and 163 eV are assigned to Cd^2+^ and S^2−^ of CdS, respectively, as shown in Figure 2(a). In Figure 2(b), the Cu_2p_ and S_2p_ peaks are observed at 940 eV and 163 eV, corresponding to Cu^1+^ and S^2−^ of Cu2S. These results confirm that the obtained samples are composed of CdS-MWNT and Cu_2_S-MWNT.

In order to obtain the composition of QDs, we measured the elemental content of the QD-MWNT supports using ICP-AES. Table 1 shows the elements present in the QD-MWNT supports. The result confirmed that the supports were successfully prepared. As mentioned above, the formation mechanism of CdS nanoparticles was described in a previous paper [32]. It is expected that copper sulfide particles are formed by the following mechanism. While the aqueous solution containing thiosulfate and metal ion is irradiated by a Co-60 γ-ray source, the thiosulfate ion in the solution is excited. These excited thiosulfate ions act as a reductant to release solvated electrons and sulfur atoms. The released solvated electrons react with sulfur atom to form sulfide ions. The metal ions then combine with sulfide ions to form sulfide particles in the irradiated region:

(1) $$S_{2}{O_{3}}^{2-}+\gamma \text{-ray}\to S+{{\text{SO}}_{3}}^{2-}$$

(2) $${{2S}_{2}O_{3}}^{2-}+\gamma \text{-ray}\to {S_{4}O_{6}}^{2-}+{2e}^{-}$$

(3) $${2\text{Cu}}^{2+}+S+{4e}^{-}\to {\text{Cu}}_{2}S$$

(4) $${\text{Cu}}^{2+}S+{2e}^{-}\to \text{CuS}$$

### 3.2. Optimization of the Prepared Microbial Biosensor and Determination of the Total Amount of Phenolics in Commercial Red Wines

**Figure 3.**
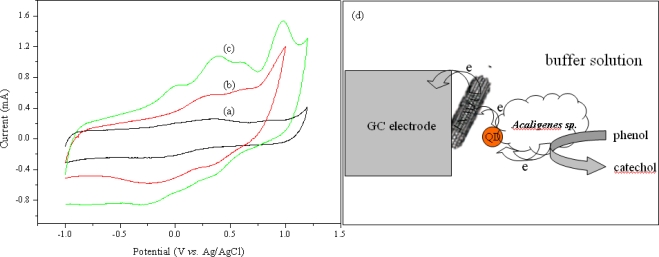
Cyclic voltammogram of microbial biosensor based on PVBA-MWNTs **(a)**, CdS-MWNT **(b)**, Cu_2_S-MWNT **(c)** for 3.0 mM phenol in 0.1 M phosphate buffer solution (pH = 7.0), and possible mechanism of electron transfer in an electrocatalytic process **(d)**.

To improve the sensitivity of the electrochemical biosensor, we synthesized the QD-MWNT supports by a one-step radiation reaction. Subsequently, we fabricated the microbial biosensor based on QD-MWNT supports, as described in Scheme 1. Figure 3 shows the cyclic voltammograms of the microbial biosensor based on PVBA-MWNT (a), CdS-MWNT (b), Cu_2_S-MWNT (c) for 3.0 mM phenol in 0.1 M phosphate buffer solution (pH = 7.0) and the possible mechanism of electron transfer in an electrocatalytic process (d). No oxidation and reduction peaks were detected for the *Acaligense sp.*-immobilized biosensor based on polymer-*g*-MWNT supports as seen in Figure 3(a). However, the microbial biosensor based on QD-MWNT supports exhibits two oxidation peaks at +0.5 V and +0.7 V and a low reduction peak, as shown in Figure 3(b) and Figure 3(c) for phenol in a phosphate solution. These peaks indicate the production of catechol or *o*-quinone from the microbial reaction in a phosphate solution. Basically, *Acaligense sp.* catalyzes the conversion of phenolic substrates to catechol and then *o*-quinone as follows:

(5) $$\text{phenol}+\mathrm{Acaligense sp.}\;\left(O_{2}\right)\to \text{catecohol}$$

(6) $$\text{catechol}+\;\mathrm{Acaligense sp.}\;\left(O_{2}\right)\to o\text{-quinone}+H_{2}O$$

(7) $$o\text{-quinone}+H^{+}+{2e}^{-}\to \text{catechol}$$

**Figure 4.**
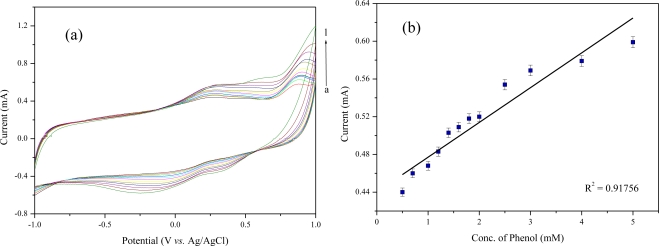
Cyclic voltammograms of the microbial biosensor based on CdS-MWNT for phenol in 0.1 M phosphate buffer solution (pH—7.0) **(a)** calibration curve of phenol concentration with respect to the current **(b)**.

**Figure 5.**
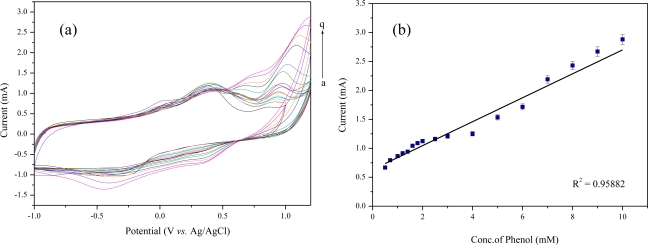
Cyclic voltammograms of microbial biosensor based on Cu_2_S-MWNT for phenol in 0.1 M phosphate buffer solution (pH—7.0) **(a)** calibration curve of phenol concentration with respect to current **(b)**.

The electrochemical biosensing of phenol was performed under optimized experimental conditions. Figures 4 and 5 show the cyclic voltammograms of phenol on the *Acaligense sp.*-immobilized biosensor based on CdS-MWNT and Cu_2_S-MWNT supports in 0.1 M phosphate buffer solution (pH = 7.0) as a function of phenol concentration. When using the biosensor prepared by CdS-MWNT, the sensing range of phenol was from 0.5 to 5.0 mM as shown in Figure 4, whereas the sensing range of the *Acaligense sp.*-immobilized biosensor based on the Cu_2_S-MWNT support for phenol was in the range of 0.7 ∼ 10 mM as shown in Figure 5.

**Figure 6.**
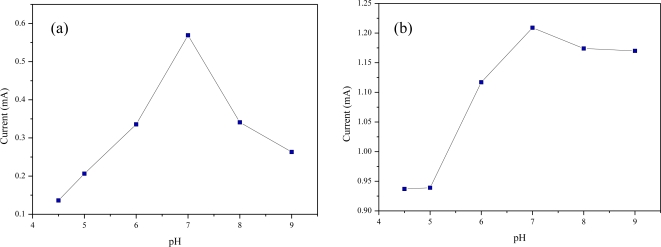
Effect of pH on the microbial biosensor based on CdS-MWNT **(a)** and Cu_2_S-MWNT **(b)** supports for 3.0 mM phenol in 0.1 M phosphate buffer solution.

Another parameter affecting the sensing efficiency of the biosensor based on the QD-MWNT supports is the pH of the supporting electrolyte. Figure 6 shows the cyclic voltammograms of 3.0 mM phenol on the *Acaligense sp.*-immobilized biosensor based on CdS-MWNT support (a), Cu_2_S-MWNT support (b) 3.0 mM phenol in 0.1 M phosphate buffer solution as a function of pH. The sensing efficiency increases with increasing pH values from 4.0 to 7.0, and then decreases with increasing pH values as shown in Figure 6(a,b). This means that at low pH values, the sensing efficiency of biosensor is not changed due to the enzyme activity. In contrast, when using pH > 7.0, the sensing efficiency rapidly decreases.

The stability of the prepared biosensors were examined by determining 3.0 mM phenol in 0.1 M phosphate buffer solution (pH = 7.0). The prepared sensors were stored in the 0.1 M phosphate buffer (pH 7.0) in a refrigerator when not in use. After 10 days of storage, both biosensors lost less than 5% of their original response.

Two red wines, namely Amor made in Chile and Blue Nun made in Germany, were used in the analysis of phenolic compounds in wines, as described in Table 2. The sensing using the *Acaligense sp.*-immobilized biosensor based on QD-MWNT supports is performed directly on 2.0 mL red wine samples. The obtained current from the red wines was compared with that obtained from phenol solution as shown in Figure 4 and 5.

## 4. Conclusions

In this study, we fabricated an *Acaligense sp*.-immobilized biosensor based on CdS-MWNT and Cu_2_S-MWNT supports prepared by a one-step radiation reaction. The sensing range of the *Acaligense sp*.-immobilized biosensor based on CdS-MWNT and Cu_2_S-MWNT supports for phenol was in the range of 0.5 ∼ 5.0 mM and 0.7 ∼ 10 mM, respectively. Both biosensors exhibited a wide linear range, high sensitivity, and good stability. The prepared biosensors were used to the determination of phenolics in commercial red wines. The results showed that the amount of phenolic compounds in commercial red wines were in the range of 926.1 ∼ 1,018 mg/L, calculated from the calibration curve of phenol measured by the *Acaligense sp*.-immobilized biosensor based on QD-MWNT supports as shown in Figures 4 and 5. The relatively high amounts of phenolic compounds in Blue Nun are responsible for the bitter taste of the red win.

## Figures and Tables

**Table 1.** Content of elements on CdS-MWNT and Cu_2_S-MWNT prepared by γ-irradiation.

**Samples**	**Cd content**	**Cu content**	**S content**
CdS-MWNTs	19.4 wt-%	-	5.9 wt-%
Cu_2_S-MWNTs	-	10.2 wt-%	5.1 wt-%

Metal content (wt-%) was determined by ICP-AES.

**Table 2.** Total amount of phenolic compounds in commercial red wines determined by the microbial sensor based on the QD-MWNT supports.

**Commercial wines**	**CdS-MWNTs ^(a)^**		**Cu_2_S-MWNTs ^(b)^**	
	**Current density**	**Phenolics**	**Current density**	**Phenolics**
Amor (Chile)	0.513 mA	926.1 mg/L	0.844 mA	1,031 mg/L
Blue Nun (Germany)	0.519 mA	948.4 mg/L	0.829 mA	1,018 mg/L

(a) The amounts of total phenolics were calculated from the calibration curve as shown in Figure 4.

(b) The amounts of total phenolics were calculated from the calibration curve as shown in Figure 5.
